# Case Report: Thymoma-associated stiff person syndrome and myasthenia gravis: an index case with exploratory exome sequencing and review of reported cases

**DOI:** 10.3389/fonc.2026.1801960

**Published:** 2026-05-01

**Authors:** Lu Zhao, Linan Fang, Mingbo Tang, Kewei Zhang, Wei Liu, Min Qiang

**Affiliations:** 1 Department of Thoracic Surgery, The First Hospital of Jilin University, Changchun, China; 2 College of Clinical Medicine, Jilin University, Changchun, China

**Keywords:** anti-glutamic acid decarboxylase, myasthenia gravis, stiff person syndrome, thymoma, tumor resection, whole-exome sequencing

## Abstract

**Background:**

Thymoma is frequently associated with myasthenia gravis (MG), but the coexisting of stiff person syndrome (SPS) is exceedingly rare. This overlap syndrome poses diagnostic and therapeutic challenges, and its immunopathogenic basis is not well understood.

**Methods:**

A 55-year-old woman with thymoma-associated SPS and MG was evaluated using detailed clinical, electrophysiological, serological and radiologic investigations. In addition, a PubMed-based literature review of published cases of thymoma complicated by SPS with MG was conducted. Exploratory whole-exome sequencing (WES) with pathway analysis were performed on resected thymoma tissue.

**Results:**

The index patient presented with progressive gait unsteadiness, painful axial and lower-limb stiffness, dysarthria and dysphagia. Examination revealed marked truncal rigidity and exaggerated startle responses. Serology demonstrated high-titer anti–glutamic acid decarboxylase 65 (GAD65) and elevated acetylcholine receptor (AChR) antibodies. Chest computed tomography (CT) demonstrated an anterior mediastinal soft-tissue mass highly suggestive of thymoma. Treatment with immunotherapy, symptomatic agents and thymectomy resulted in substantial clinical improvement. The literature search identified seven additional patients with thymoma-associated SPS and MG, typically characterized by GAD65 positivity, World Health Organization (WHO) B1/B2 mixed histology, and favorable outcomes after combined immunotherapy and surgical resection. WES of the thymoma revealed a somatic CACNA1A frameshift variant and variants in immune-signaling genes, with enrichment of pathways related to calcium channels, γ-aminobutyric acidergic (GABAergic) synapses and immune regulation.

**Conclusion:**

Thymoma-associated SPS and MG constitute a rare but recognizable overlap syndrome. Integration of clinical, literature and exploratory genomic data supports a convergent mechanism involving thymic epithelial neoplasia, impaired inhibitory neurotransmission and neuromuscular junction autoimmunity.

## 1 Introduction

Thymoma is a neoplasm arising from thymic epithelial cells and typically exhibits low malignant potential. It predominantly occurs in the anterior superior mediastinum, with peak incidence between 45 and 60 years of age (1, 2). The thymus normally functions as the primary organ for T-cell maturation and central immune tolerance induction in childhood and early adulthood. By adolescence, the thymus typically begins to involute physiologically. The main pathological types of the thymus include thymic hyperplasia (characterized by lymph follicular expansion, often associated with autoimmune diseases), thymoma (neoplastic proliferation of thymic epithelial cells with varying degrees of lymphocyte infiltration), and thymic atrophy (age-related or stress-mediated degeneration of thymic tissue). Beyond its local compressive effects- such as chest tightness, cough, and chest pain- thymoma is clinically significant because of its ability to disrupt central immune tolerance. This dysregulation underlies a broad spectrum of paraneoplastic syndromes, among which the association with myasthenia gravis (MG) is the most prominent. Approximately 30-70% of patients with thymoma developed MG, while 10-30% of MG cases harbor an underlying thymoma (3–5). Both conditions can be diagnosed concurrently, or MG may occur years after tumor resection. Thymoma appears to exert dual immunoregulatory effects: aberrant expression of self-antigens within thymic epithelial cells may promote the generation of pathogenic autoantibodies, whereas altered thymic microarchitecture may also modulate autoimmune activity in a subset of patients (4, 5). These features pose significant perioperative challenges, especially among patients with generalized MG who are at increased risk for postoperative respiratory complications, thereby necessitating meticulous preoperative optimization and intra-/postoperative monitoring.

MG is an autoimmune disorder of neuromuscular transmission characterized by fluctuating skeletal muscle weakness and fatigability (6). Symptoms such as diplopia and ptosis often worsen throughout the day (4). Pathophysiologically, autoantibodies against the acetylcholine receptors (AChR) impair synaptic transmission and may expand over time from ocular involvement to generalized disease affecting bulbar, axial, limb, and respiratory muscles (1). Diagnosis requires a combination of clinical assessment, serological testing for AChR antibodies, pharmacologic testing, and electrophysiologic studies (1, 4). While immunomodulatory therapies, including corticosteroids, steroid-sparing agents, and cholinesterase inhibitors, form the cornerstone of long-term management, the application of complement inhibitors and neonatal Fc receptor (FcRn) antagonists are increasing. Therapies such as plasma exchange (PLEX) and intravenous immunoglobulin (IVIG) are the main means to address acute exacerbations and myasthenia gravis crises, and thymectomy remains a key component of care in patients with thymoma (4, 7).

Stiff-person syndrome (SPS) is a rare autoimmune neurologic disorder characterized by progressive rigidity and episodic, often painful spasms of the axial and proximal limb muscles. Symptoms are typically triggered or exacerbated by emotional stress, tactile stimuli, or sudden auditory input. SPS is strongly associated with antibodies glutamic acid decarboxylase 65 (GAD65), leading to impaired γ-aminobutyric acid (GABA) synthesis and disruption of inhibitory neurotransmission (8–10). Although most cases are autoimmune and idiopathic, SPS can occur as a paraneoplastic syndrome associated with malignant tumors, such as breast cancer, lung cancer, and certain lymphomas (11). Genetic testing, including panel sequencing and whole exome sequencing (WES), is generally reserved for atypical presentations (10). The current evidence suggests that SPS arises from a complex interaction between autoimmunity and polygenic susceptibility rather than a single- gene defect.

While the association between thymoma and MG is well established, the coexistence of SPS with thymoma is exceedingly rare (12). The combined occurrence of MG and SPS in patients with thymoma implies that thymic epithelial tumors may create an immunologic environment capable of generating multiple, anatomically distinct autoimmune responses. Aberrant antigen expression and impaired negative selection within the neoplastic thymus may promote autoantibody formation against both peripheral neuromuscular junction components and central GABAergic targets. This triad thus represents a mechanistic framework for exploring how thymic neoplasia orchestrates parallel autoimmune processes across the peripheral and central nervous systems.

## 2 Methods

### 2.1 Index case: clinical and diagnostic evaluation

This study was conducted in accordance with the Declaration of Helsinki and was approved by the institutional ethics committee. Written informed consent for publication was obtained from the patient.

A 55-year-old woman was admitted to the Department of Neurology with a 2-week course of fluctuating lower-limb symptoms characterized by progressive gait unsteadiness, painful stiffness of the lower limbs and trunk, dysarthria and dysphagia. A detailed clinical history was obtained, including comorbidities and medication exposure. Neurological examination focused on muscle tone, strength, reflexes, coordination, sensory function and gait.

Routine blood tests included complete blood count, electrolytes, liver and kidney function tests, thyroid function and inflammatory markers. Brain and spinal magnetic resonance imaging (MRI) were performed to exclude structural lesions. Electrophysiological studies comprised electromyography (EMG) and repetitive nerve stimulation, in accordance with standard protocols.

Serologic testing included GAD65 antibodies, AChR-IgG and other neuronal antibodies (e.g. anti-glycine receptor, anti-amphiphysin, ganglioside antibodies) when available. SPS was diagnosed based on characteristic axial and limb stiffness, painful spasms, exaggerated startle and compatible serology or EMG findings. MG was diagnosed based on fluctuating fatigable weakness, compatible neurophysiological testing and elevated AChR antibodies.

Chest computed tomography (CT) was used to screen for anterior mediastinal lesions. Thymoma was confirmed by histopathological examination of the resected tumor and classified according to the World Health Organization (WHO) histological classification.

### 2.2 Literature review of thymoma-associated SPS and MG

A literature search was conducted to identify reported cases of thymoma complicated by SPS and MG. PubMed was searched from inception to November 2025. The retrieval terms were composed of the Medical Subject Headings (Mesh) database: (“Thymoma” [Mesh] OR thymoma [tiab]) AND (“Stiff-Person Syndrome” [Mesh] OR stiff person [tiab] OR stiff-person [tiab] OR SPS [tiab] OR “stiff-person spectrum” [tiab]) AND (“Myasthenia Gravis” [Mesh] OR myasthenia gravis [tiab] OR MG [tiab]). Only English-language reports were included. Inclusion criteria were: (1) pathologically confirmed thymoma; (2) clinical diagnosis of SPS based on typical stiffness and spasms with supportive serological and/or electropgysiological findings; and (3) documented MG with serologic or neurophysiological confirmation where available. Reports lacking sufficent clinical detail, without confirmed thymoma, or without clear SPS and MG diagnosis were excluded. For each eligible case, data were extracted on age, sex, sequence of SPS and MG onset, antibody profiles, thymoma histology, treatments (immunotherapy, symptomatic drugs, thymectomy) and clinical outcomes. Cases were summarized descriptively and tabulated.

### 2.3 Tumor whole-exome sequencing and bioinformatic analysis

Given the unusual overlap of SPS and MG in the context of a thymic epithelial tumor and the relapsing course after surgery, we performed WES of the resected tumor with tumor-only exploratory WES.

Raw reads were trimmed and aligned to the human reference genome (GRCh37/hg19). Variant calling was performed using a standard pipeline with quality filtering. Nonsynonymous single-nucleotide variants, splice-site alterations and small insertions/deletions with adequate coverage and high-quality scores were retained for annotation. Variant annotation included gene function and known disease associations.

Pathway enrichment analysis was conducted using curated databases such as Kyoto Encyclopedia of Genes and Genomes (KEGG) or Reactome to explore over-represented pathways in the set of candidate genes, with particular attention to synaptic, calcium signaling and immune-related pathways. Protein–protein interaction (PPI) networks were constructed using the STRING database to visualize interactions among selected genes of interest, including CACNA1A, guanine nucleotide exchange factor 2 (VAV2), integrin alpha L (ITGAL), mitogen-activated protein kinase kinase 2 (MAP2K2) and intercellular adhesion molecule 1 (ICAM1). Given the single-case design and absence of matched normal tissue, all genomic findings are interpreted as hypothesis-generating.

## 3 Results

### 3.1 Index case: clinical course, treatment and outcome

#### 3.1.1 Clinical presentation

The 55-year-old woman had a past history of newly diagnosed hypertension (1 week) and hypothyroidism (7 years). The patient presented with a 2-week course of fluctuating lower-limb symptoms characterized by progressive stiffness and weakness, painful spasms, and recurrent falls. Over the preceding 2 weeks, she developed dysarthria, dysphagia, markedly reduced bilateral pharyngeal reflexes, horizontal nystagmus in both eyes, increased muscle tone in the left lower limb, and decreased superficial sensation in the lower limbs. One month before symptom onset, the patient had self-administered levofloxacin and clarithromycin for an upper respiratory infection.

On neurological examination, dysarthria and dysphagia were noted. Gaze-evoked horizontal nystagmus was elicited on bilateral abduction and was absent in primary gaze. Muscle strength in all four limbs was preserved (Medical Research Council [MRC] grade 5/5); however, muscle tone was noticeably increased in the left lower limb. Deep tendon reflexes were normal and symmetrical in all extremities, and plantar responses were flexor bilaterally with no pathological reflexes. Babinski and Chaddock signs were negative bilaterally. Dysarthria and dysphagia were clinically consistent with a flaccid bulbar pattern. The patient reported mild fatigability with slight worsening toward the evening, suggesting diurnal fluctuation consistent with myasthenic involvement. Superficial sensation in the lower limbs was decreased, whereas deep sensation (joint position and vibration) was preserved. The finger-to-nose test on the right side was slightly unsteady and inaccurate. The heel-knee-shin test of the left lower limb could not be performed reliably because of marked stiffness and weakness. Romberg’s test could not be adequately assessed, as the patient was unable to maintain an upright stance safely.

#### 3.1.2 Diagnostic investigations

Routine laboratory investigations were unremarkable or showed only minor nonspecific changes. Brain and spine MRI showed no structural abnormalities. EMG showed no abnormal spontaneous activity or continuous motor unit firing. No findings suggestive of characteristic agonist–antagonist co-contraction were observed. Repetitive nerve stimulation was performed but did not demonstrate a significant decremental response. Single-fiber EMG was not performed.

Serologic testing revealed positive anti-GAD65 antibodies (titer 1:32) and elevated AChR-IgG levels of 3.52 nmol/L. In our reference laboratory, the starting dilution is 1:10 for serum and 1:1 for cerebrospinal fluid; serum anti-GAD65 antibody titers <1:10 are interpreted as negative and serum AChR-IgG levels below 0.5 nmol/L are considered negative. A serum titer of 1:10 is considered weakly positive, and 1:32 corresponds to a moderately positive result. The patient’s AChR-IgG level was 3.52 nmol/L, which was markedly above the reference range (<0.5 nmol/L), supporting a true-positive result rather than incidental low-titer positivity. Given the complex neurological phenotype and suspected autoimmune/paraneoplastic involvement, extended antibody screening (including anti-ganglioside antibodies) was performed. Peripheral nerve antibody screening demonstrated positivity for IgM antibodies against GM1 and GM3 (GM1/GM3 IgM) and IgM antibodies against GD1b (GD1b IgM). Cerebrospinal fluid analysis showed positivity for glycine receptor alpha-1 (GlyRα1) antibodies (titer 1:3.2) and anti–GAD65 antibodies (titer 1:10). The combination of clinical features and antibody profile supported diagnoses of SPS and MG in the setting of suspected paraneoplastic autoimmunity.

**Figure 1:**
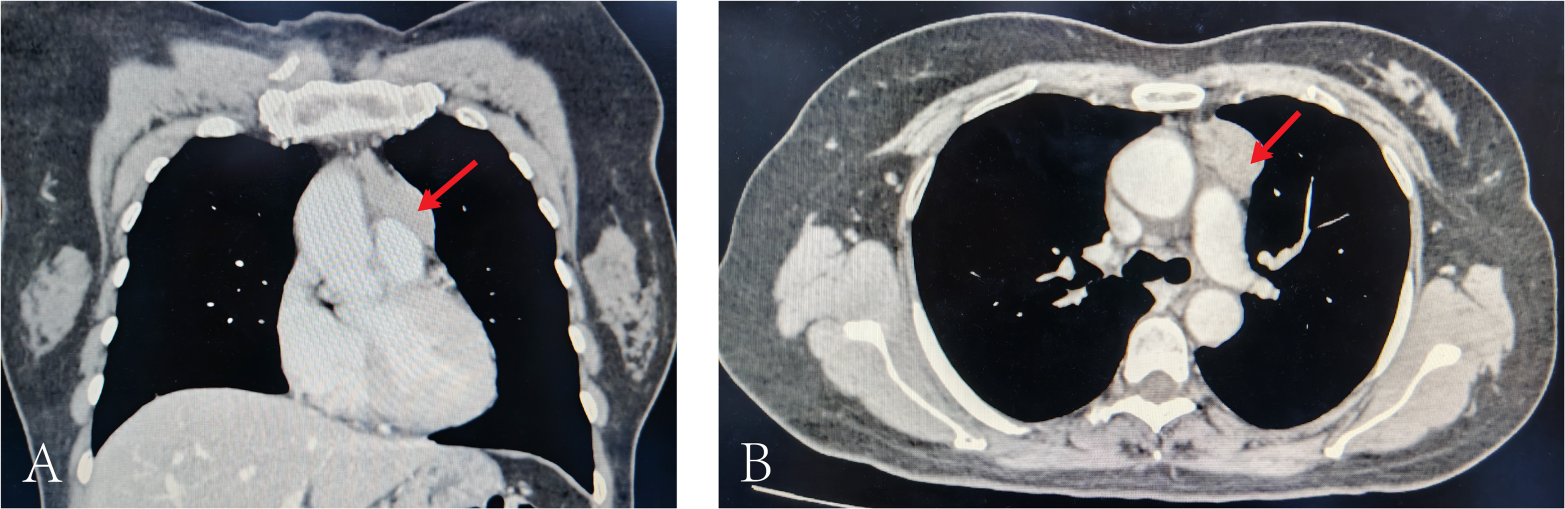
Contrast-enhanced chest CT demonstrating thymoma. **(A)** Coronal reconstruction. **(B)** Sagittal reconstruction. Red arrows indicate the anterior mediastinal mass consistent with thymoma.

Chest CT demonstrated an anterior mediastinal soft-tissue mass measuring approximately 3.5 × 2.4 cm, highly suggestive of a thymic epithelial tumor (**Figure 1**). There was no radiological evidence of distant metastasis on CT/Positron Emission Tomography–Computed Tomography (PET/CT).

#### 3.1.3 Treatment and outcome

The immediate postoperative course was uneventful. At early reassessment, the symptoms of SPS remained stable, with marked improvement in dysphagia and dysarthria. The AChR-IgG titer had also fallen from 3.52 to 0.47 nmol/L. After adequate control of spasms and pain, the patient underwent extended thymectomy via median sternotomy with complete resection of the mediastinal mass and surrounding thymic tissue.

**Figure 2:**
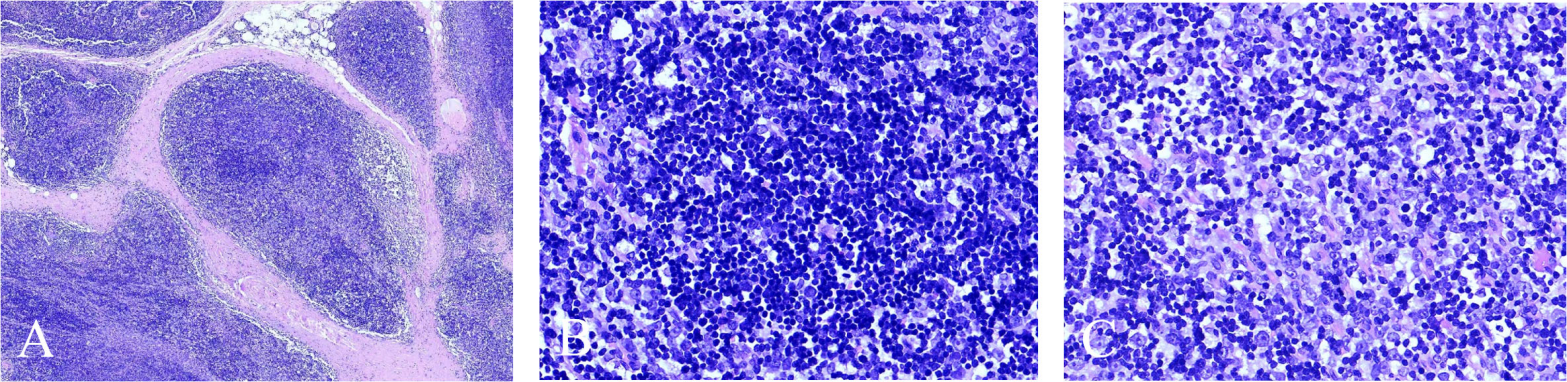
Histopathological features of thymoma (WHO type B1/B2 mixed). **(A)** Low-power view showing fibrous septa subdividing the tumor into variably sized lobules with prominent lymphocytic infiltration; focal areas resemble normal thymic medullary architecture (×40). **(B)** Representative WHO type B1 component: medulla-like areas with abundant non-neoplastic (reactive) immature T lymphocytes and scattered neoplastic thymic epithelial cells with bland cytomorphology (oval to spindle nuclei, fine chromatin, inconspicuous or small nucleoli, mild atypia, scant pale cytoplasm) (×400). **(C)** Representative WHO type B2 component: approximately equal proportions of neoplastic thymic epithelial cells and non-neoplastic (reactive) immature T lymphocytes; epithelial cells are larger, round to polygonal, with enlarged oval nuclei, vesicular chromatin, and conspicuous nucleoli (×400).

Histopathological examination of the resected anterior mediastinal mass revealed a thymoma, classified as WHO type B1/B2 mixed (**Figure 2**). The tumor measured 4.5 × 3.5 × 1.8 cm and showed focal invasion into the surrounding capsular adipose tissue, without definite vascular or perineural invasion. Two perithymic lymph nodes and one separately submitted level-6 lymph node were negative for tumor (0/2 and 0/1, respectively). Immunohistochemical staining demonstrated that the tumor cells were positive for p63, pan-cytokeratin (AE1/AE3), and CAM5.2, while TdT, CD1a, CD5, CD3, and CD20 were negative. P53 showed a wild-type pattern, and Ki-67 staining was positive, indicating a proliferative tumor cell population.

### 3.2 Early postoperative relapse and follow-up evaluation

Three weeks after thymectomy, the patient was readmitted because of generalized stiffness and spasms, severe pain, and a subjective “frozen” sensation in the lower limbs, resulting in multiple falls from an upright position. On examination, the patient was fully conscious and able to communicate. Muscle strength remained normal (MRC 5/5), but muscle tone was markedly increased, and even mild stimuli elicited painful tonic–clonic spasms. Superficial sensation in the lower limbs was reduced, whereas deep sensation was preserved. Repeat serologic testing demonstrated persistently positive anti–GAD65 IgG antibodies (titer 1:100). Peripheral nerve antibody screening again revealed positivity for GM1/GM3 IgM and GD1b IgM, with additional weak positivity for GM4-IgM and sulfatide-IgM. Follow-up chest CT and whole-body PET/CT showed no new or recurrent lesions.

The patient received corticosteroids, intravenous immunoglobulin (IVIG), antispasmodic therapy to reduce muscle tone, neurotrophic agents, analgesics, and general supportive care. A physiatry-supervised rehabilitation program was initiated to promote gait training and functional recovery. After a three-week course of treatment, she showed marked clinical improvement with substantial functional recovery.

### 3.3 Literature review of thymoma-associated SPS and MG

The literature search identified seven previously reported patients with thymoma-associated SPS and MG that met the inclusion criteria. Together with the index case, a total of eight patients were analyzed (**Table 1**).

**Table 1:** Reported cases of thymoma with coexisting stiff-person spectrum disorder (SPS) and myasthenia gravis (MG) (published cases and the present case).

First author	Age	Sex	Clinical course	Anti-AchR ab	Anti-GAD ab	WHO classification	Year
Nicholas (17)	55	M	SPS→thymectomy→MG	–	–	B2	1997
Tanaka (15)	57	F	SPS→thymectomy→MG	–	+	B1	2005
Thomas (18)	45	M	SPS→thymectomy→MG	-→ +	+	NR	2005
Morise (19)	72	F	MG→SPS→ thymectomy	+	+	B2	2015
Lee (20)	48	F	MG→thymectomy→SPS	+	+	B1	2017
Mehta (21)	30	M	SPS, MG→thymectomy	+	+	NR	2020
Okado (12)	74	M	SPS, MG→thymectomy	+	+	B2	2025
Our case	55	F	SPS, MG→thymectomy	+	+	B1&B2	2025

SPS, Stiff person syndrome; MG, Myasthenia gravis; anti-AchR ab, Anti-acetylcholine receptor antibody; Anti-GAD ab, Anti glutamic acid decarboxylase antibody; Not performed thymectomy; NR, Not recorded; WHO, World Health Organization.

Across these eight patients, age at onset ranged from 30 to 74 years, with a balanced sex distribution (four males and four females). In most reports, SPS manifestations (truncal stiffness, painful spasms and exaggerated startle) preceded or occurred in close temporal proximity to MG symptoms; in a minority of cases, MG was the initial presentation followed by SPS. Clinical courses clustered into three patterns: SPS preceding thymectomy then MG (3/7); MG preceding SPS (1/7); and concurrent SPS-MG before surgery (3/7); one additional case progressed MG preceding SPS (Progressive Encephalomyelitis with Rigidity and Myoclonus, PERM) then thymectomy (counted within the “MG then SPS before surgery” group).

Serologically, anti-AChR antibodies were reported as positive in the majority of patients, and anti-GAD antibodies were positive in all but one case. Where thymoma histology was documented, all tumors were classified as WHO type B1 or B2, indicating lymphocyte-rich thymic epithelial tumors was B1 in 2/7, B2 in 3/7, and 2 older reports did not specify histological subtype. Our patient had a B1/B2 mixed thymoma. Year of publication ranged from 1997 to 2025, underscoring that this overlap syndrome has been sporadically recognized over nearly three decades.

Therapeutically, all patients received immunotherapy in various combinations, most frequently corticosteroids, intravenous immunoglobulin and/or plasma exchange, together with symptomatic agents such as benzodiazepines and baclofen for stiffness and spasms. Thymectomy was performed in all but one older report, and was temporally associated with improvement or stabilization of both SPS and MG manifestations in most cases. Nevertheless, some patients experienced incomplete remission or relapse, emphasizing that autoimmunity may persist despite tumor removal once central tolerance has been disrupted. Overall, the collated data support thymoma-associated SPS and MG as a rare but clinically consistent overlap syndrome, characterized by middle- to older-age onset, frequent anti-GAD and anti-AChR positivity and B1/B2-type thymoma, with a generally favorable but sometimes incomplete response to combined immunotherapy and thymectomy.

### 3.4 Exploratory tumor exome findings

Exploratory WES of the resected thymoma generated high-quality sequencing data. WES did not reveal canonical druggable oncogenic drivers but confirmed microsatellite stability (MSS) and low tumor mutational burden (TMB), and identified, among these, a somatic truncating mutation in ATR and a frameshift deletion in CACNA1A (c.3411del), the latter mapping to the GABAergic synapse pathway. CACNA1A (c.3411del) predicted to result in truncation of the Cav2.1 P/Q-type voltage-gated calcium channel α1A subunit, lacking part of the C-terminal region important for channel modulation and synaptic vesicle release. Variants were also observed in immune-related genes such as ITGAL (integrin αL), MAP2K2 (mitogen-activated protein kinase kinase 2 [MEK2]), VAV2 (a guanine nucleotide exchange factor involved in cytoskeletal dynamics and T-cell receptor signaling) and (intercellular adhesion molecule 1, a key mediator of leukocyte adhesion and transmigration).

Exploratory pathway analysis (given tumor-only sequenceing limitations) suggested over-representation of pathways related to synaptic function, calcium signaling and immune regulation, including GABAergic synapse–associated components and cytokine-receptor interaction. No pathogenic germline variants were inferred due to lack of matched control. PPI network analysis using STRING incorporating CACNA1A, ITGAL, MAP2K2 and VAV2 yielded a statistically significant interaction network (P < 0.001), highlighting potential convergence between calcium channel function and immune signaling pathways.

These genomic observations, although preliminary and derived from a single tumor sample without matched normal tissue, are compatible with a hypothesis that thymoma-driven alterations in calcium channel–related genes and immune signaling may contribute to the breakdown of tolerance to neuronal antigens such as GAD65 and neuromuscular junction components such as AChR.

## 4 Discussion

Thymomas represents a unique epithelial neoplasm with profound immunological implications, capable of generating both local mass effects and systemic autoimmune manifestations. Its well-established association with MG arises from disordered thymic architecture, impaired negative selection, and aberrant self-antigen presentation, which permit escape of autoreactive T-cell clones (5, 13, 14). In contrast, the co-occurrence of SPS, a rare GABAergic autoimmune encephalomyelitis, is distinctly uncommon, accounting for only a small fraction of paraneoplastic SPS cases (15). The simultaneous emergence of thymoma, MG, and SPS—as illustrated by the present case—highlights a convergence of immune dysregulation affecting both the neuromuscular junction and the inhibitory pathways of the central nervous system.

SPS is principally mediated by autoantibodies targeting GAD65, resulting in impaired GABA synthesis and hyperexcitability of axial and proximal limb musculature. Approximately 60% of patients exhibit high-titer GAD65 antibodies (15), while a subset also harbors antibodies against GABA receptor–associated proteins (16). MG, by contrast, arises from autoantibody-mediated disruption of AChR signaling at the neuromuscular junction (1, 4). The coexistence of these two distinct autoimmune neurologic phenotypes in patients with thymoma suggests a shared upstream immune disturbance emanating from the tumor microenvironment.

A literature review identified seven previously published cases of thymoma-associated SPS and MG (12, 13, 17–21). It is consistent with the known propensity of these histologic subtypes to disrupt thymic epithelial organization and promote autoreactive T-cell export (13, 22, 23). Several individuals demonstrated clinical exacerbation or unmasking of MG or SPS following thymectomy, necessitating IVIG or plasma exchange—underscoring the need for peri-operative vigilance in thymoma-related neuroimmunologic disorders.

In this case, symptom fluctuations may have been potentiated by medication exposures. Fluoroquinolones and macrolides—both taken by the patient shortly before symptom onset—are recognized precipitants of MG exacerbation, with fluoroquinolones carrying an Food and Drug Administration (FDA) black-box warning (24, 25). Calcium channel blockers, including levamlodipine, have also been implicated as potential neuromuscular transmission inhibitors (26). Collectively, these agents may have amplified underlying autoimmune susceptibility, accelerating clinical deterioration.

To explore potential genetic contributors, WES was performed on thymoma tissue. It revealed a somatic CACNA1A frameshift variant and rare variants affecting several immune-signaling genes- VAV2, ITGAL, MAP2K2, ICAM1. CACNA1A encodes the α1A subunit of P/Q-type voltage-gated calcium channels, which mediate presynaptic calcium entry and neurotransmitter release (27–30). Loss-of-function mutations can induce cerebellar dysfunction, dystonia, and impaired acetylcholine release at the neuromuscular junction (28–30). VAV2, a member of VAV family of guanine nucleotide exchange factors (GEFs), is an important regulator of immune signaling ^31.^ In T cells, VAV2 can dampen T-cell receptor (TCR)–induced Ca²^+^ influx via Cdc42, thereby potentially gating activation thresholds and stabilizing the immunological synapse; however, these observations derive largely from murine and cell-based models, with limited direct human evidence (31, 32). ITGAL, also call CD11a, governs lymphocyte adhesion and contributes to cancer progression and the tumor immune microenvironment (33). MAP2K2 is a dual-specificity kinase within the Extracellular signal-regulated kinase (ERK) pathway and is essential for growth factor–driven cell-cycle progression (34). ICAM1 is central to lymphocyte–endothelial adhesion and broader immune cell–cell interactions (T cell–T cell and T cell–B cell), participating in the regulation of immune responses and cell-mediated cytotoxicity (35).

PPI analysis yielded a highly significant integrated network, supporting integrating GABAergic-pathway genes with these four immune-related genes (P < 0.001). Taken together, these findings point to a convergent disturbance of GABAergic signaling and immune regulation in this case. The CACNA1A alteration may represent an upstream perturbation within this network, although causality cannot be inferred from a single case.

Single-cell RNA-sequencing data from thymic epithelial tumors have shown that medullary thymic epithelial cells (mTECs) can ectopically express GAD65 under Autoimmune Regulator (AIRE)-positive conditions (36). Although CACNA1A is normally expressed at very low levels in the thymus, with only rare cells co-expressing CACNA1A and GAD2 (36). Taken together, these findings support a mechanistic hypothesis in which a somatic CACNA1A loss-of-function mutation arising within a GAD65-expressing thymic epithelial clone disrupts local GABAergic signaling and antigen processing, impairing central tolerance to GAD65, AChR, and related synaptic antigens. Such a model offers a biologically plausible explanation for the simultaneous development of SPS and MG in the context of thymoma.

Overall, this case underscores the remarkable immunopathogenic breadth of thymoma—capable of inducing multifocal autoimmunity across both peripheral and central nervous systems. Integration of clinical observations, literature data, and exploratory genomic analysis suggests a convergent mechanism involving thymic epithelial neoplasia, aberrant antigen presentation, and dual disruption of GABAergic and cholinergic synaptic systems. Further mechanistic studies, particularly those leveraging single-cell genomics and thymic microenvironment models, are warranted to clarify how specific somatic events in thymoma initiate such complex neuroimmunologic phenotypes.

## 5 Conclusion

Thymoma can precipitate complex paraneoplastic autoimmune neuromuscular syndromes, including the rare co-occurrence of SPS and MG. This case highlights the need to consider overlapping autoimmunity in patients with thymic epithelial tumors presenting with both stiffness and fatigable weakness. Prompt recognition, coordinated multidisciplinary care, and timely oncologic management are pivotal to optimizing neurologic and oncologic outcomes.

## Data Availability

The datasets presented in this study can be found in online repositories. The names of the repository/repositories and accession number(s) can be found in the article/supplementary material.
